# Metanálise Pré-clínica: Outro Tijolo na Parede

**DOI:** 10.36660/abc.20200551

**Published:** 2020-11-01

**Affiliations:** 1 Universidade Estadual Paulista “Júlio de Mesquita Filho” Faculdade de Medicina BotucatuSP Brasil Universidade Estadual Paulista “Júlio de Mesquita Filho” Faculdade de Medicina - Campus de Botucatu, Botucatu, SP - Brasil; 2 Universidade Estadual Paulista “Júlio de Mesquita Filho” Faculdade de Medicina BotucatuSP Brasil Universidade Estadual Paulista “Júlio de Mesquita Filho” Faculdade de Medicina - Campus de Botucatu - Clínica Médica, Botucatu, SP - Brasil

**Keywords:** Metanálise, Revisão Sistemática, Publicações Científicas, Interpretação Estatística de Dados, Heterogeneidade

Atualmente, a revisão sistemática e a metanálise são consideradas o nível 1 de evidência científica, sendo amplamente utilizadas em epidemiologia e na medicina baseada em evidências. O termo metanálise vem do grego e significa “avaliação de análises”, referindo-se a sua definição como “análise estatística de uma grande coleção de resultados de análises de estudos individuais com o objetivo de integrar os achados” proposta pelo estatístico Gene V. Glass em 1976.^1^

Apesar das semelhanças, é importante notar que metanálise e a revisão sistemática não são sinônimos. A metanálise é frequentemente, mas nem sempre, precedida por uma revisão sistemática, que visa reunir estudos semelhantes que atendam aos critérios de elegibilidade de abordagem rígida para responder a uma questão de pesquisa específica, enquanto a metanálise integra os resultados dos estudos incluídos através de técnicas estatísticas.^2^^,^^3^

Basicamente, quando conduzida de forma adequada, a grande vantagem de uma meta-análise é o poder estatístico substancialmente aumentado, que fornece uma estimativa precisa do tamanho do efeito de um conjunto de estudos, sendo considerada a melhor evidência disponível.^4^ Além disso, ela pode ser aplicável a amplo espectro de tópicos, incluindo biomarcadores, fatores genéticos, diagnóstico e tratamento.^4^^,^^5^

Por outro lado, as principais críticas às metanálises dizem respeito à heterogeneidade entre os estudos e ao viés de publicação, ambos comprometendo a robustez e a validação dos resultados.^6^ É inevitável que haja diversidade de desenhos, intervenções, exposições e resultados em uma coleção de estudos; no entanto, é essencial quantificar a extensão da heterogeneidade realizando abordagens estatísticas, como análise de sensibilidade, subgrupo ou regressão.^4^ Além disso, o “*file drawer problem*” (ou o “problema da gaveta de arquivo”) ou seja, estudos menores ou com resultados negativos ou não significativos tendem a permanecer não publicados, e isso pode superestimar o grau do efeito real levando ao viés de publicação. Podemos avaliar o viés de publicação em metanálise utilizando método de amostra denominado gráfico de funil (*funnel plot*) ou outro método estatístico, como o teste de regressão de Egger método *Trim-and-Fill*, a depender do caso, também disponível para este propósito.^3^^,^^7^^–^^9^

Tradicionalmente, as meta-análises são realizadas com dados de estudos em humanos. Entretanto, nos últimos anos, a utilização de metanálises de estudos pré-clínicos tem se tornado mais frequente. Várias razões estão relacionadas à falta de estudos clínicos sobre um assunto específico, especialmente quando se trata de tópicos que não são possíveis de serem investigados em humanos, enquanto alguns modelos animais já são bem padronizados.^10^

Neste sentido, edição recente dos Arquivos Brasileiros de Cardiologia publicou interessante metanálise sobre o efeito do exercício aeróbico na prevenção de disfunção cardíaca em murinos expostos à doxorrubicina.^11^ Novos conhecimentos nessa área são sempre bem-vindos, uma vez que a cardiotoxicidade induzida pela doxorrubicina é uma das principais consequências graves de seu uso e, atualmente, as terapias para prevenir ou atenuar a cardiotoxicidade são escassas e ineficazes.

Em resumo, os autores demonstraram que a prática de exercícios aeróbicos contribuiu para melhorar a pressão desenvolvida e a fração de encurtamento do ventrículo esquerdo em murinos com disfunção cardíaca causada pelo tratamento com doxorrubicina. Certamente, é uma conclusão muito importante, pois o exercício aeróbico pode ser uma boa estratégia não farmacológica para prevenir, atenuar ou tratar a cardiotoxicidade, uma vez que não tem (ou tem mínimos) efeitos colaterais, além de poder trazer benefícios adicionais para a saúde humana.

Vale ressaltar que os estudos em animais são frequentemente pequenos e inerentemente heterogêneos e, portanto, são obrigados a seguir abordagem metodológica rígida.^10^ A presente metanálise foi bem conduzida e seguiu todas as recomendações atuais para a sua preparação. Entretanto, ela traz alguns pontos que devem ser levados em consideração para aumentar a qualidade dos resultados e sua interpretação.

Tópico importante a ser destacado é que os estudos que compõem esta metanálise mostraram grandes diferenças na dose de doxorrubicina, no momento em que o exercício foi implementado (pré ou pós-exposição à doxorrubicina), na modalidade de exercício e intensidade do exercício, fatores esses que conferem uma grande heterogeneidade entre os estudos (I^2^ = 87 e 94%). Esse é um problema frequente em metanálises realizadas com estudos experimentais. É possível minimizar a heterogeneidade incluindo algumas análises de subgrupos ou aspectos limitantes do desenho do estudo durante a estratégia de busca. Além disso, como descrito anteriormente, é muito difícil publicar resultados negativos, principalmente em pesquisas experimentais, o que poderia atribuir viés de publicação a este estudo.

Em nossa opinião, a principal vantagem de qualquer metanálise é o fornecimento de evidências científicas robustas para o desenvolvimento de diretrizes que apoiem os profissionais de saúde a tomarem uma decisão clínica ideal. Obviamente, produzir evidências científicas para diretrizes clínicas não deve ser o objetivo de metanálises conduzidas com estudos pré-clínicos. Elas são classicamente exploratórias e nos permitem gerar hipóteses que podem ser utilizadas para projetar e conduzir futuros ensaios clínicos. Elas oferecem a possibilidade de incluir mais um tijolo no muro do conhecimento científico.
