# Supplementary material for: Metanálise Pré-clínica: Outro Tijolo na Parede
Source: Arq Bras Cardiol. 2020 Nov 1;115(5):894–5. [Article in Portuguese] doi: 10.36660/abc.20200551 (PMC8452194; doi:10.36660/abc.20200551)
Supplement: Translation

# Translation: Pre-Clinical Meta-Analysis: Another Brick in the Wall

**Affiliations:** 1 BotucatuSP Brazil Universidade Estadual Paulista “Júlio de Mesquita Filho” Faculdade de Medicina - Campus de Botucatu, Botucatu, SP - Brazil; 2 BotucatuSP Brazil Universidade Estadual Paulista “Júlio de Mesquita Filho” Faculdade de Medicina - Campus de Botucatu - Clínica Médica, Botucatu, SP - Brazil

**Keywords:** Meta-Analysis, Systematic Review, Scientific Publications, Data Interpretation, Statistical

Currently, systematic review and meta-analysis are considered level 1 of scientific evidence and it is widely used in epidemiology and evidence-based medicine. The term meta-analysis comes from the Greek and means “analysis of analyses”, referring to its definition “statistical analysis of a large collection of analysis results from individual studies for the purpose of integrating the findings” proposed by the statistician Gene V. Glass in 1976.^1^

Despite the similarities, it is important to note that meta-analysis and systematic review are not synonymous. Meta-analysis is often, but not always, preceded by a systematic review, which aim to gather similar studies that meet the strict approach eligibility criteria in order to answer a specific research question, whereas meta-analysis integrates the results of included studies by statistical techniques.^2^^,^^3^

Basically, when conducted properly, the major advantage of a meta-analysis is the statistical power substantially increased, which provides a precise estimate of the effect size of a set of studies, being considered the best evidence available.^4^ Besides, can be applicable to a broad spectrum of topics, including biomarkers, genetic factors, diagnosis, and treatment.^4^^,^^5^

On the other hand, the main criticism regarding meta-analyzes are about heterogeneity among the studies and publication bias, both compromising the robustness and the validation of results.^6^ Undoubtedly, it is inevitable a diversity of design, interventions, exposures and outcomes in a collection of studies; however, it is essential to quantify the extent of heterogeneity by performing statistical approaches such as sensitivity, subgroup, or regression analyses.^4^ In addition, the “file drawer problem”, that is smaller studies or with negative or non-significant results tend to remain unpublished and this might overestimate the actual effect degree leading to publication bias. We can assess publication bias in a meta-analysis using a sample method called funnel plot or another statistical method depending on the case, such as Egger's regression test, trim and fill method, also available for this purpose.^3^^,^^7^^–^^9^

Traditionally, meta-analyzes are performed of data from human studies. However, in the last years, the use of meta-analysis from preclinical studies has become more frequent. Several reasons are related to the lack of clinical studies about a specific issue, especially when dealing with topics that are not possible to investigate in humans, whereas optimal methods on animal models are largely standardized.^10^

In this sense, a recent issue of the *Arquivos Brasileiros de Cardiologia* published an interesting meta-analysis about the effect of aerobic exercise on the prevention of cardiac dysfunction in murines exposed to doxorubicin.^11^ New knowledge in this field is always welcome, since doxorubicin-induced cardiotoxicity is one of the main serious consequences of its use and currently therapies to prevent or attenuate cardiotoxicity are scarce and not effective.

In brief, the authors have showed that the practice of aerobic exercise contributed to improve left ventricle fractional shortening and left ventricle developed pressure in murines with cardiac dysfunction caused by doxorubicin treatment. Certainly, it is a very important conclusion because the aerobic exercise can be a good non-pharmacological strategy to prevent, to attenuate or to treat cardiotoxicity, since it has no (or minimum) side effects and could has additional benefits for human healthy.

It is worth to mentioning that animal studies are frequently small and inherently heterogeneous and, therefore, are required to follow all the strict methodological approach.^10^ The present meta-analysis was well conducted and followed all the current recommendations for its preparation. However, it brings up some points that should be taken into account, in order to increase the quality of the results and their interpretation.

The important topic to be highlighted is that studies that compound this meta-analysis shown great differences in the dose of doxorubicin, moment that exercise was implemented (pre or post doxorubicin exposure), modality of exercise and intensity of exercise, which attribute a wide heterogeneity between the studies (I^2^= 87 and 94%). It is a frequent problem in meta-analysis carried out experimental studies. It is possible to minimize the heterogeneity including some subgroups analysis or limiting study design aspects during the search strategy. Additionally, as previously described, it is very difficult to publish negative results, mainly in experimental researches, which could attribute publication bias to this study.

In our opinion, the mainly advantage of meta-analysis is provided robust scientific-based evidence for developing guidelines that supports health care professionals to make an optimal clinical decision. Obviously, producing scientific evidence for clinical guidelines should not be the purpose of meta-analysis conducted with pre-clinical studies. They are classically exploratory and allow us generate hypothesis that can be used to design and conduct future clinical trials. They offer the possibility to include one more brick in the wall of scientific knowledge.
